# Results of Behavioral Evaluations Predict Length of Stay for Shelter Dogs

**DOI:** 10.3390/ani11113272

**Published:** 2021-11-16

**Authors:** Betty McGuire, Jordan Chan, Kennedy Jean-Baptiste, Philippa Kok, Emma Rosenbaum

**Affiliations:** 1 Department of Ecology and Evolutionary Biology, Cornell University, Ithaca, NY 14853, USA; jc2598@cornell.edu (J.C.); err73@cornell.edu (E.R.); 2 Department of Animal Science, Cornell University, Ithaca, NY 14853, USA; kaj95@cornell.edu (K.J.-B.); pvk8@cornell.edu (P.K.)

**Keywords:** dog, animal shelter, behavioral evaluation, length of stay, adoption

## Abstract

**Simple Summary:**

It is common practice for animal shelters to evaluate the behavior of dogs a few days after admission. These evaluations typically consist of a series of tests and subtests that expose dogs to diverse stimuli and situations they might encounter postadoption. Limited information exists on whether behaviors displayed during an evaluation predict a dog’s length of stay at the shelter. We examined records from 975 dogs behaviorally evaluated and released for adoption at a New York shelter. Proportions of the study population evaluated as displaying concerning or especially dangerous behavior on tests and subtests were generally low. Nevertheless, dogs’ scores on some tests or subtests (food guarding and meeting another dog) predicted length of stay at the shelter. Dogs evaluated as showing dangerous behavior had longer lengths of stay than dogs evaluated as showing either concerning behavior or no concerning behavior; the latter two groups did not differ from one another in length of stay. We suggest that dogs with challenging behaviors have smaller pools of potential adopters, which leads to longer lengths of stay. Our findings may aid shelter management of dog populations and help highlight dogs needing special adoption efforts to avoid long stays at shelters.

**Abstract:**

Most animal shelters conduct behavioral evaluations before making dogs available for adoption. However, little information exists on whether behaviors displayed during these assessments predict a dog’s length of stay at the shelter. We reviewed nearly 5 years of records from 975 dogs released for adoption at a New York shelter to see whether behaviors shown during their evaluation predicted length of stay. For most tests and subtests, the prevalence of concerning and especially dangerous behaviors was low. Nevertheless, dogs’ scores on some tests or subtests—food guarding and meeting another dog—predicted length of stay at the shelter. Dogs evaluated as showing dangerous behavior had longer lengths of stay than dogs evaluated as showing either concerning behavior or no concerning behavior; the latter two groups did not differ from one another in length of stay. The most likely explanation for the relationships found between behavior during the evaluation and length of stay at the shelter is that dogs with challenging behaviors had smaller pools of potential adopters. Understanding the relationships between performance on behavioral evaluations and length of stay may inform shelter management of canine populations and also help identify dogs requiring special adoption efforts to avoid long shelter stays.

## 1. Introduction

There is much debate over the usefulness of canine behavioral evaluations conducted at animal shelters. These evaluations typically occur several days after a dog has been admitted to the shelter and include a series of tests and subtests meant to assess a dog’s reaction to diverse stimuli and situations. Proponents of behavioral evaluations see them as useful, when combined with other sources of information, in promoting better informed matches between dogs and adopters and helping to prevent dangerous dogs from entering communities [1,2]. Critics describe behavioral evaluations as provocative and unlikely to reliably predict behavior postadoption, especially because obviously dangerous dogs are usually screened out of the population at or shortly after intake (i.e., before the behavioral evaluation), resulting in a low prevalence of warning or biting behaviors in the evaluated dog population [3,4]. Additionally, critics suggest time and resources would be better spent engaging with dogs in positive activities they will experience in adoptive homes, such as training, taking walks on a leash, and playing with other dogs [3]. Several studies have surveyed adopters of shelter dogs to determine if results from shelter behavioral evaluations predict the behavior of dogs in adoptive homes. In general, these studies have found poor predictability for behaviors such as food guarding and separation-related behaviors and somewhat better predictability for behaviors related to sociability and fearfulness [5,6,7,8,9,10,11]. Another approach taken to assess the effectiveness of shelter behavioral evaluations uses privately-owned dogs: owners complete the Canine Behavioral Assessment and Research Questionnaire (C-BARQ), a validated questionnaire [12], and then dogs are evaluated by researchers using different shelter assessments. Correlations between behavior reported by owners and behavior scored during shelter behavioral evaluations are typically weak to moderate at best [13,14].

Less explored than their predictability regarding behavior postadoption, is whether results from behavioral evaluations predict other metrics of interest to shelters, such as the percentage of adopted dogs that are returned (often called return rate) and length of stay (time from intake to adoption). Some information is available for the specific tests that assess food guarding because most shelters test for this behavior and about half of those surveyed in one study did not make food guarding dogs available for adoption [5], making it essential to understand the predictive abilities of food guarding tests. Of the dogs assessed as food guarding in shelters, most (83%) exhibit behaviors consistent with mild to moderate guarding (e.g., stiffening and growling); the remaining dogs (17%) exhibit behaviors classified as severe guarding (e.g., lunging, snapping, and biting; [15,16]). In one study, dogs that showed severe food guarding during the shelter behavioral evaluation were more likely to be returned than those that showed either mild to moderate guarding or no guarding behavior, and the latter two groups did not differ from one another in the likelihood of return [16]. Another study found slightly lower return rates for dogs that displayed food guarding during the behavioral evaluation (5%; not differentiated by level of severity) when compared with dogs that did not display food guarding (9%; [5]). However, food guarding dogs in the study by Mohan-Gibbons et al. [5] were placed on a free-feeding program in the shelter as well as on a specific feeding program in the adoptive home (although adopters did not always follow the program). Regarding length of stay, dogs that showed food guarding at the time of evaluation stayed at one shelter four days longer, on average, than dogs in the general shelter population [15]. Behavior around food, however, represents only one of the many situations and behavioral tendencies typically examined in a shelter evaluation (e.g., fearfulness, sociability, arousal during play, and responses to an unfamiliar person, unfamiliar dog, and handling). The scarcity of information concerning the relationship between behaviors displayed during shelter evaluations and length of stay is surprising given that other factors potentially influencing length of stay have been extensively studied, including canine demographic and phenotypic characteristics [17,18,19,20,21,22,23], as well as in-kennel behavior [17,21,24,25] and whether a dog is housed at the shelter or in a foster home [26].

We reviewed nearly five years of data from a New York SPCA to determine whether behavior displayed during separate tests and subtests of the shelter behavioral evaluation, together with the demographic factors sex and age, predicted length of stay for dogs. Based on previous research at this shelter regarding whether food guarding predicted the likelihood of return to the shelter [16], we predicted that dogs evaluated as showing dangerous behavior on specific tests and subtests would have longer lengths of stay than dogs evaluated as showing either concerning behavior or no concerning behavior. We expected dogs showing either concerning behavior or no concerning behavior to have similar lengths of stay. Understanding the relationship between length of stay and behavior during specific tests and subtests of canine behavioral evaluations could inform shelter management of dog populations. A second goal was to determine the prevalence of concerning or dangerous behaviors during specific tests and subtests of the behavioral evaluation because few measures of prevalence for these assessments have been reported in the literature (exceptions include [8,9]).

## 2. Materials and Methods

### 2.1. Study Shelter and Records

We examined canine records from the Tompkins County SPCA in Ithaca, NY, USA, which is a no-kill, open-admission shelter with scheduled intake. The shelter has several programs to promote dog adoptability including a small set of foster homes, playgroups for suitable pairs of dogs, and volunteer dog walking, in-kennel socialization, and taking dogs on day trips or overnight stays. Adoptions are promoted in local print and social media, at off-site events, and by a volunteer group independently advertising hard-to-place dogs.

We analyzed data from 1 September 2014 through to 31 May 2019 entered by shelter staff into the PetPoint data management system (Supplementary Material). We extracted demographic data on dogs (sex, age, and body mass), as well as information on behavioral evaluations, adoptions, and lengths of stay (date adoption paperwork signed minus intake date, in days; for dogs returned one or more times to the shelter, we used their first length of stay). We excluded records from the following groups: all puppies (behavioral evaluations of puppies differed from those of older dogs and puppy results were not entered into the PetPoint database); five dogs with serious medical conditions; and dogs returned to owners, transferred to rescue organizations, or euthanized for either medical or behavioral reasons. We also excluded records from 34 dogs released for adoption and kept in foster homes during the study period because length of stay differed between dogs housed at the shelter and in foster homes (mean ± *SD*: shelter, 19.7 ± 19.7 days; foster home, 48.4 ± 47.5 days; *t* = 3.52, *d.f.* = 33.40, *p* < 0.01). Our final sample consisted of 975 dogs released for adoption following behavioral evaluation and housed at the shelter until adoption (note that this sample essentially reflects that of McGuire [16], but without the dogs in foster homes or with serious health issues).

### 2.2. Dogs, Care, and Housing

We provide a brief description of housing and care of dogs because details have been presented by McGuire [27]. Upon admission to the shelter, dogs were housed in chain link cages in the Rescue building. Each cage had an indoor space (2.2 m^2^) and an outdoor run (3.5 m^2^). Veterinary staff examined dogs on the day of intake and performed routine procedures such as vaccinations, flea control, fecal exam, deworming, and a heartworm test. Each dog was scheduled for behavioral evaluation (Section 2.3) and after completing the behavioral evaluation, dogs were moved within a few days to the Pet Adoption Center, adjacent to the Rescue building. Thirteen cubicles on the adoption floor ranged in size from 5.2 to 7.3 m^2^ and almost all dogs were housed individually; only dogs surrendered from the same household and assessed by staff as needing to stay together shared the same cubicle. Each cubicle contained a water bowl, raised bed, blanket, and toys. Staff fed dogs each day between 08:00 and 09:00 h and between 15:00 and 16:00 h. Dogs were exercised several times a day when volunteers or staff either walked them or brought them to a large outdoor enclosure. Intact dogs were spayed or neutered before adoption.

Demographic data on the 975 dogs are summarized in Table 1. Most dogs at the Tompkins shelter were mixed breeds; due to a lack of pedigrees and DNA analyses, the number of purebred dogs in the shelter population during the study period was unknown.

### 2.3. Behavioral Evaluations

Approximately 3 days after intake, shelter staff evaluated each dog’s behavior using a series of tests based on Sternberg’s Assess-a-Pet [28], with modifications described by Bollen and Horowitz [1]. Present at each test was an evaluator from the shelter’s Behavior Program and a scribe. Over the nearly 5-year study period there were four evaluators (all female); beginning in June of 2015 and running through to the end of our study, evaluations were almost always conducted by one of the four evaluators. Behavioral evaluations included nine tests in the following sequence, with the Cage presentation subtests conducted while the dog was in its kennel in the Rescue building and all other tests and subtests conducted in a conference room in the Pet Adoption Center with the dog leashed.

1. Cage presentation (two subtests): confrontational, evaluator faces the dog, bends at the waist, and makes direct eye contact; friendly, evaluator faces sideways, bends down, and talks to the dog in friendly manner.
2. Sociability (four subtests): evaluator stands and ignores the dog for 60 s; strokes the dog three times; sits and ignores the dog for 5 s; sits and talks to the dog for 20 s.
3. Teeth exam: evaluator makes five attempts to lift the dog’s upper lip and hold for 5 s.
4. Handling (eight subtests): evaluator strokes the dog’s far side; lifts hind foot; runs hand down tail and tugs slightly; checks ears; presses on shoulders; leads with collar; wipes with towel; hugs.
5. Arousal: evaluator initiates play for 30 s using balls, rope toys, etc., and then stops.
6. Food bowl: evaluator gives the dog a mix of kibble and canned food in a bowl, and using the Assess-a-Hand, strokes the dog’s back and attempts to pull the bowl away.
7. Possession: evaluator gives the dog a valued possession such as a raw hide chew or pig’s ear, and using the Assess-a-Hand, attempts to take the item away.
8. Stranger: an unfamiliar person knocks on the door to the conference room, enters when prompted by the evaluator, makes eye contact with the dog, steps forward and reaches toward the dog, then squats and talks to the dog in a friendly manner.
9. Dog-to-dog (two subtests): with the evaluator holding the leash of the dog being tested, the scribe brings from the adoption floor a leashed dog that did not show aggression to other dogs during its own evaluation and reactions of the test dog are recorded first upon seeing and then upon meeting the previously tested dog.

Most dogs were tested with one dog, although sometimes a test dog’s reaction prompted testing with a second previously tested dog, and early in the study period, dogs with a history of dog aggression were tested with a fake dog. Use of a fake dog was largely discontinued after 2017 due to questions about the predictive value of a dog’s reaction to the fake dog compared to real dogs.

Dogs evaluated as showing concerning or dangerous responses were placed on behavior modification plans and handling plans; housing strategies were also employed (e.g., dogs evaluated as reactive to other dogs were housed in cubicles on the adoption floor that minimized exposure to other dogs). Dogs on behavior plans were not retested.

### 2.4. Scoring Methods and Statistical Analyses

Evaluators attempted to conduct all nine parts of the evaluation to their full extent, but exceptions were made for safety reasons for two tests requiring close contact. If a dog snapped at the evaluator during one of the early attempts of the Teeth exam, then subsequent attempts to check the teeth were skipped. Similarly, if a dog reacted poorly to one of the handling subtests, such as being led by the collar, then the evaluator might skip the hug subtest and proceed to other components of the evaluation. To address the reality that the Teeth exam was sometimes cut short and certain subtests of the Handling test may have been skipped, we scored whether a dog showed concerning or dangerous behavior during *at least one attempt* of the Teeth exam and during *at least one subtest* of the Handling test.

Table A1 lists by test the behaviors considered concerning or dangerous by evaluators. For most tests in which a sufficient number of dogs showed concerning or dangerous behavior, we considered the level of behavior in our analyses using the following categories: no concerning behavior; concerning behavior; and dangerous behavior (Teeth exam; Handling; Stranger; Dog-to-dog meeting; an exception was made for the Food bowl test and Possession test, see below). Dangerous behaviors were not listed in the shelter scoring system for the four subtests of the Sociability test and the seeing subtest of the Dog-to-dog test because none of these subtests created situations in which dangerous behaviors were displayed. Thus, for Sociability subtests and the Dog-to-dog seeing subtest, we present data for concerning behaviors only. Finally, for the Cage presentation confrontational and friendly subtests, we present data for dangerous behaviors only, due to differences between the options available on the shelter behavioral evaluation form for concerning behaviors and entry options in PetPoint.

To be consistent with previously published research, we categorized data from the Food bowl test and Possession test somewhat differently than the above descriptions for other tests. First, studies of resource guarding in shelter dogs typically combine results from these two tests [6,7,16], so we combined them in our analyses as well. Second, previous studies classified the level of guarding based on behaviors shown during either the food bowl test, possession test, or both tests as follows: dogs that stiffened, exhibited whale eye, snarled, froze, or growled were classified as showing mild to moderate guarding, and dogs that lunged, snapped, or bit the Assess-a-Hand were classified as showing severe guarding [15,16]. Note that this system of scoring differs somewhat from the shelter scoring system in that only the last three behaviors considered dangerous at the shelter are classified as severe guarding (lunged, snapped, bit the Assess-a-Hand; Table A1), and the behaviors froze and growled, classified by the shelter as dangerous, are classified as mild to moderate guarding, along with stiffened, exhibited whale eye, and snarled.

For each test on the behavioral evaluation, we first assessed the prevalence of dogs in the study population showing concerning or dangerous behavior (number of dogs showing concerning or dangerous behavior/number of dogs tested and released for adoption). For tests with a sufficient number of dogs showing concerning or dangerous behavior, we used least squares models to determine significant predictors of length of stay at the shelter. Fixed factors in the models for length of stay were sex, age class, and behavior during the specific test or subtest (e.g., no concerning behavior, concerning behavior, or dangerous behavior). For all models, we examined the main effects and two-way interactions. From the final models, we dropped two-way interactions that were not significant at the *p* < 0.05 level; none of our two-way interactions were significant, thus all were dropped. We used Tukey’s HSD to correct for multiple comparisons within models. To control for multiple testing across models and decrease the likelihood of false positives, we set the *p* value threshold at *p* ≤ 0.01. Statistical analyses were completed in JMP Pro (version 15.0.0). For subtests or tests with a very low prevalence of concerning or dangerous behaviors (Cage presentation friendly subtest and Arousal test), we provide descriptive statistics only because models were unstable.

## 3. Results

### 3.1. Prevalence

Prevalence of dogs evaluated as showing concerning or dangerous behavior on each test or subtest of the behavioral evaluation is shown in Table 2. Of all the tests and subtests, we found the highest prevalence for showing concerning behavior during the stand and ignore portion of the Sociability test (34.7%; only concerning behaviors scored, no dangerous behaviors), meaning that it was fairly common for dogs to make very brief nonsocial contact with the evaluator or to completely ignore the evaluator during this subtest. The prevalence of dogs evaluated as showing dangerous behavior during tests ranged from 0.0% (Arousal test) to 4.1% (Cage presentation confrontational subtest; Table 2). For the Food bowl and Possession tests, 12.0% of dogs showed mild to moderate guarding and 2.6% of dogs showed severe guarding (these results are reported here rather than in Table 2 because of slight differences in how we categorized behaviors).

### 3.2. Length of Stay

For ease of presentation, we first provide descriptive statistics for length of stay at the shelter in relation to demographic variables (Table 3) and then in relation to the behavior displayed during specific tests and subtests of the behavioral evaluation (Table 4, tests for which the level of behavior could be analyzed using the categories: no concerning behavior, concerning behavior, or dangerous behavior; Table 5, subtests for which only concerning behaviors were scored). Because we used a slightly different scoring system for food guarding, we describe these results in the text rather than including them in Table 4. The specific results of statistical models incorporating both demographic variables and behavior during the test or subtest are summarized in Table 6.

Sex did not predict length of stay of dogs at the shelter (Table 3 and Table 6). In contrast, age class did predict length of shelter stay: seniors stayed longer than adults, which in turn stayed longer than juveniles (Table 3 and Table 6).

**Table 3:** Length of stay (mean ± *SD*, in days) for shelter dogs placed up for adoption in relation to sex and age class, with sample sizes in parentheses. Within specific variables, values with different superscript letters are significantly different.

Demographic Variables	Length of Stay (Days)
Sex	
Female	18.7 ± 20.6 (479)
Male	20.6 ± 18.9 (496)
Age class	
Juvenile	14.8 ± 11.6 (183) ^a^
Adult	19.8 ± 21.1 (674) ^b^
Senior	26.4 ± 19.9 (118) ^c^

Level of behavior displayed by dogs on some tests/subtests predicted length of stay at the shelter. For the Dog-to-dog meeting subtest, dogs evaluated as showing dangerous behavior had longer lengths of stay than dogs evaluated as showing either concerning behavior or no concerning behavior; the latter two groups did not differ from one another in length of stay (Table 4 and Table 6). Although results for the Teeth exam, Handling test, and Stranger test showed a similar pattern, the effect of level of behavior on length of stay failed to reach statistical significance at the *p* ≤ 0.01 level (Table 4 and Table 6). Level of food guarding behavior displayed during either the Food bowl test, Possession test, or both tests predicted length of stay at the shelter (Table 6). Dogs evaluated as showing severe guarding had longer lengths of stay (mean ± *SD*, in days, number of dogs; 34.2 ± 20.2, 25) than dogs evaluated as showing either mild to moderate guarding (20.6 ± 13.8, 117) or no guarding behavior (19.1 ± 20.3, 833); the latter two groups did not differ from one another in length of stay.

**Table 4:** Length of stay (mean ± *SD*, in days) for dogs in relation to behavior shown during tests or subtests on the behavioral evaluation at the shelter. Number of dogs shown in parentheses. Within rows, values with different superscript letters are significantly different.

Test	No Concerning Behavior	Concerning Behavior	Dangerous Behavior
Teeth exam	19.4 ± 19.9 (940)	21.8 ± 11.1 (16)	31.1 ± 15.4 (19)
Handling	19.2 ± 20.1 (836)	21.5 ± 17.3 (113)	26.9 ± 16.4 (26)
Stranger	19.6 ± 19.6 (919)	16.4 ± 13.5 (31)	29.0 ± 27.2 (25)
Dog-to-dog: meeting	19.0 ± 19.3 (829) ^a^	22.2 ± 17.8 (130) ^a^	36.7 ± 41.3 (16) ^b^

For the Sociability test in which only concerning behaviors were scored by shelter staff, behavior during the four subtests did not predict length of stay at the shelter: length of stay did not differ between dogs that showed concerning behavior and dogs that did not (Table 5 and Table 6). In contrast, there was a trend (*p* = 0.012) for behavior during the seeing component of the Dog-to-dog test to predict length of stay at the shelter. The post-hoc comparison revealed that dogs evaluated as showing concerning behavior had longer lengths of stay than dogs evaluated as showing no concerning behavior (Table 5 and Table 6).

**Table 5:** Length of stay (mean ± *SD*, in days) for dogs in relation to behavior shown during the four subtests of the Sociability test and the seeing subtest of the Dog-to-dog test. Number of dogs shown in parentheses. Within rows, values with different superscript letters are significantly different ^1^.

Test	No Concerning Behavior	Concerning Behavior
Sociability: stand and ignore	19.0 ± 20.8 (637)	21.0 ± 17.6 (338)
Sociability: three strokes	19.6 ± 20.0 (910)	20.3 ± 16.1 (65)
Sociability: sit and ignore	19.1 ± 19.9 (802)	22.3 ± 18.9 (173)
Sociability: pet and talk	19.7 ± 19.8 (948)	21.0 ± 19.8 (27)
Dog-to-dog: seeing	19.4 ± 19.0 (942) ^a^	28.4 ± 34.6 (33) ^b^

^1^ Only concerning behaviors were scored because these subtests do not create situations in which dangerous behaviors occur.

Behavior during the Cage presentation confrontational subtest (only dangerous behaviors scored) did not predict length of stay at the shelter: dogs that showed dangerous behavior did not differ in length of stay from those that did not show dangerous behavior (mean ± *SD*, in days, number of dogs; dangerous behavior, 20.2 ± 15.3, 40; no dangerous behavior, 19.7 ± 19.9, 935; Table 6). We present descriptive statistics only for length of stay for the Cage presentation friendly subtest (only dangerous behaviors scored) and the Arousal test because models were unstable due to the small number of dogs showing dangerous or concerning behavior during these two tests (Cage presentation friendly subtest: dangerous behavior, 16.3 ± 10.3, 16; no dangerous behavior, 19.8 ± 19.9, 959; Arousal test: concerning behavior, 24.7 ± 14.8, 16; no concerning behavior, 19.6 ± 19.8, 959; even though dangerous behaviors were possible during the Arousal test, none of the dogs tested displayed them).

**Table 6:** Results of least squares models to determine predictors of length of stay of dogs at the shelter. Fixed factors are sex, age class, and behavior displayed during the specific test or subtest. Threshold for significance set at *p* ≤ 0.01 ^1^.

Test	Source	*d.f.*	*F*	*p*
Cage presentation: confrontational	Sex	1	2.12	0.15
	Age class	2	12.65	0.0001
	Behavior during subtest ^2^	1	0.03	0.87
Sociability: stand and ignore	Sex	1	2.25	0.14
	Age class	2	11.91	0.0001
	Behavior during subtest ^3^	1	0.96	0.33
Sociability: three strokes	Sex	1	2.16	0.15
	Age class	2	12.64	0.0001
	Behavior during subtest ^3^	1	0.10	0.76
Sociability: sit and ignore	Sex	1	2.38	0.13
	Age class	2	11.84	0.0001
	Behavior during subtest ^3^	1	2.33	0.13
Sociability: pet and talk	Sex	1	2.12	0.15
	Age class	2	12.63	0.0001
	Behavior during subtest ^3^	1	0.07	0.79
Teeth exam	Sex	1	2.35	0.13
	Age class	2	12.71	0.0001
	Behavior during test ^4^	2	3.55	0.03
Handling	Sex	1	1.73	0.19
	Age class	2	13.24	0.0001
	Behavior during test ^4^	2	2.82	0.06
Food bowl and Possession	Sex	1	1.85	0.17
	Age class	2	11.52	0.0001
	Behavior during tests ^4^	2	5.98	0.003
Stranger	Sex	1	2.36	0.13
	Age class	2	12.67	0.0001
	Behavior during test ^4^	2	3.37	0.04
Dog-to-dog: seeing	Sex	1	1.97	0.16
	Age class	2	12.56	0.0001
	Behavior during subtest ^3^	1	6.38	0.012
Dog-to-dog: meeting	Sex	1	2.86	0.09
	Age class	2	11.74	0.0001
	Behavior during subtest ^4^	2	7.23	0.001

^1^ Results are not shown for the Cage presentation friendly subtest and the Arousal test because few dogs showed dangerous or concerning behaviors during testing and least squares models were unstable. ^2^ Only dangerous behaviors were analyzed for the Cage presentation confrontational subtest because options available on the shelter behavioral evaluation form for concerning behaviors differed from entry options in PetPoint. ^3^ Only concerning behaviors were analyzed for the four Sociability subtests and the seeing component of the Dog-to-dog test because these subtests do not create situations in which dangerous behaviors occur. ^4^ For three tests (Teeth exam, Handling, and Stranger) and one subtest (Dog-to-dog: meeting) we classified behavior by the following levels: no concerning behavior, concerning behavior, or dangerous behavior. For the Food Bowl and Possession tests, we used the following levels to be consistent with existing literature: no guarding behavior, mild to moderate guarding, or severe guarding.

## 4. Discussion

A relatively low prevalence of concerning and especially dangerous behaviors characterized our study population of 975 dogs evaluated and released for adoption. The Sociability stand and ignore subtest had the highest prevalence at 34.7%, indicating that about one third of dogs tested and made available for adoption showed concerning behavior during this subtest, defined as ignoring or making only brief social contact with the evaluator. The prevalence of dangerous behaviors in our study population never exceeded 4.1% on any of the tests and subtests in which such behaviors might be displayed.

It is challenging to compare the prevalence of behaviors during shelter behavioral evaluations across different studies because of variation in the tests and subtests conducted and scoring systems used. For example, whereas the evaluation at our study shelter classified behaviors displayed by dogs as dangerous, concerning, or not concerning, other studies classified behaviors into categories such as fearful, anxious, or aggressive [8,9]. A few direct comparisons are possible, with the caveat, that even tests with the same or similar names may be conducted and scored differently at different shelters. The prevalence of food guarding among dogs placed up for adoption at our study shelter (overall, 14.6%) is similar to values reported for other shelters (14% [5]; 20.6% [6]; 17% [15]). Clay et al. [8] reported 9.9% of dogs guarded a bone and 7.4% guarded a pig’s ear, but specific data on guarding a food bowl were not included. Despite testing for food guarding, van der Borg et al. [9] did not report the prevalence of this behavior. These authors did, however, report that 32.1% of dogs tested showed aggressive behavior (described as growling, baring teeth, snapping, biting, and piloerection) when meeting another dog. In contrast, we found the prevalence of concerning behavior (13.3%) and dangerous behavior (1.6%) during the Dog-to dog meeting subtest to be lower than that reported by van der Borg et al. [9]. This might reflect different testing conditions: whereas van der Borg et al. [9] matched dogs for sex and size, and described the dogs used for testing as dominant, these conditions did not apply to the Dog-to-dog subtests at our study shelter. Finally, the prevalence of concerning behavior (1.6%) and dangerous behavior (0.0%) during the Arousal test at our study shelter was also lower than the 12.3% of dogs reported by van der Borg et al. [9] to show either aggressive responses or play escalating into aggression during the Play with handler test.

Level of behavior displayed by dogs on some tests or subtests predicted length of stay at the shelter. When using the levels, dangerous behavior, concerning behavior, and no concerning behavior, the dogs evaluated as showing dangerous behavior during the Dog-to-dog meeting subtest had longer lengths of stay (on average, about 15 days longer) than dogs that showed either concerning behavior or no concerning behavior. We found no difference in length of stay between dogs evaluated as showing concerning behavior and those evaluated as showing no concerning behavior during the Dog-to-dog meeting subtest. The level of food guarding behavior, assessed as severe, mild to moderate, or no guarding during either the Food bowl test, Possession test, or both tests, also predicted length of stay at the shelter. Dogs evaluated as showing severe guarding had longer lengths of stay (on average, about 14 days longer) than dogs evaluated as showing either mild to moderate guarding or no guarding; there was no difference in length of stay between dogs that showed either mild to moderate guarding or no guarding. These length of stay results for the Dog-to-dog meeting subtest and food guarding tests mirror those described by McGuire [16]. For likelihood of return of food guarding dogs to this shelter, dogs evaluated as showing severe guarding were more likely to be returned than those evaluated as showing either mild to moderate guarding or no guarding, and the latter two groups did not differ in likelihood of return. It is worth noting, however, that even though scores on food guarding tests predicted length of stay (present study) and likelihood of return [16] at our study shelter, food guarding during shelter testing did not consistently signal such guarding would occur in adoptive homes. Surveys of adopters revealed that more than half of the dogs evaluated as food guarding at our study shelter did not show guarding postadoption [7]; similar findings have been reported for dogs at other shelters [5,6].

When levels of behavior were limited to concerning behavior or no concerning behavior for subtests that did not create situations in which dangerous behavior might be displayed, there was a trend (*p* < 0.012) for the level of behavior during the Dog-to-dog seeing subtest to predict length of stay. More specifically, dogs evaluated as showing concerning behavior remained at the shelter longer (on average, about nine days longer) than those evaluated as not showing concerning behavior. The remaining tests or subtests either did not predict length of stay (Cage presentation confrontational subtest, four Sociability subtests, Teeth exam, Handling test, and Stranger test) or too few dogs displayed concerning or dangerous behaviors to allow a formal statistical analysis (Cage presentation friendly subtest and Arousal test). Protopopova et al. [25] identified several in-kennel behaviors that predicted longer lengths of stay at the shelter; these included leaning on kennel walls, facing away from the front of the kennel, and standing. Given this connection between a dog’s in-kennel presentation and length of stay, we were surprised to find that the Cage presentation confrontational subtest, a subtest for which only dangerous behaviors were analyzed, did not predict length of stay at our study shelter. One possible explanation for our failure to find a relationship between behavior during this subtest and length of stay concerns the cage design. Cages in the Rescue building, where this test is conducted, are chain link, allowing the evaluator to bend and look directly into the eyes of the dog. In contrast, in the Pet Adoption Center, where potential adopters typically view dogs, the lower parts of cubicle doors and walls are covered with opaque material, making it impossible for visitors to bend and look directly into the eyes of dogs. Thus, the cage design in the Pet Adoption Center may reduce the occurrence of strong responses by dogs to visitors, and could explain our failure to find a relationship between behavior during the Cage presentation confrontational subtest and length of stay.

The longer lengths of stay found for dogs evaluated as displaying dangerous or concerning behavior during specific tests and subtests of the behavioral evaluation may reflect a smaller pool of potential adopters for dogs with challenging behaviors. A reduction in the size of the pool of potential adopters could happen in several ways. It is possible that potential adopters might skip over dogs with signage on their cubicle indicating issues such as food guarding or intolerance of other dogs. Another possibility is that potential adopters decline to adopt based on their direct interactions with a dog when introduced by shelter staff or volunteers. Visitors to one animal shelter stopped to look at less than one third of available dogs in their kennels, and when they did stop, spent 70 s, on average, in front of a cage [29]. Removal of breed labels from kennel cards was associated with reduced lengths of stay for dogs at one shelter, but other changes during the study period, such as increased advertising and longer operating hours, likely also played a role [18]. Another study found that most potential adopters asked to interact with only one dog and interactions with the dog were typically of short duration, about 8 min [30]. Nevertheless, dogs that were adopted spent more time lying in proximity to the adopter and less time ignoring play initiation by the adopter than dogs that were not adopted [30].

Based on these findings regarding visitor behavior at other shelters, we suggest that visitor response to signage or direct interactions with dogs probably play some role in limiting the pool of potential adopters for dogs with behavioral challenges. Our study shelter does not conduct policy-based adoptions, described in Weiss et al. [31] as screening potential adopters based on factors such as daily time spent away from home and veterinary care provided to pets already in the household. Instead, Tompkins County SPCA uses a conversation-based approach during which adoption counselors disclose all available information about the dog, including results from behavioral evaluations, to help potential adopters decide whether the dog would be a good fit for their household. Some potential adopters may decide that a dog with challenging behaviors would not be a good match for them, which might result in longer shelter stays for these dogs compared with dogs without such challenges. Tompkins County SPCA does require a meeting between any dog(s) living in the household of potential adopters and the dog they are considering bringing home. These dog meets might be another point at which potential adopters decide not to pursue a dog with behavioral challenges and could explain the relationships we found between the behavior shown during Dog-to-dog tests and length of stay. Based on the data available to us, however, we cannot assess the relative importance of these various factors—signage, direct interactions with dogs, meetings with adoption counselors, and dog meets—in the decision made by visitors to adopt or not adopt.

For the two demographic variables examined, we found that age class predicted length of stay at the shelter, but sex did not. More specifically, seniors stayed longer than adults (on average, by about 7 days), which in turn, stayed longer than juveniles (on average, by about 5 days). Our finding of increasing length of stay with age agrees with results from several studies [19,22,23]. Protopopova et al. [17] found that age did not predict length of stay at their study shelter and Luescher and Medlock [32] found age did not predict the likelihood of adoption; these findings might reflect the absence from both studies of dogs more than 7 years of age. For comparison, senior dogs, defined as those at least 8 years of age, made up about 12% of our study population. No consistent pattern has emerged from studies examining the effects of sex on length of stay: some, like ours, report no effect of sex, whereas others find an effect, often with longer stays for males [17,19,21,22,23].

Our study focused on a single shelter rather than multiple shelters in different regions, which may reduce the generalizability of our results to other shelters [20,33]. Other limitations of our study derive largely from the necessary safety measures taken by shelter staff conducting the behavioral evaluations. For example, we could not examine individual subtests of the Handling exam because reactions of a dog during one subtest may cause the evaluator to skip another subtest for safety reasons. As a result, we could not assess the prevalence of dogs showing concerning or dangerous behavior for each of the eight subtests, which ranged from touching the back foot to hugging the dog, nor could we determine whether individual subtests predicted length of stay. Instead, our more general analysis considered whether showing concerning or dangerous behavior on at least one subtest of the Handling exam affected length of stay. A similar situation existed for the Teeth exam where all five attempts to check the teeth might not have been completed for safety reasons. Another limitation concerns variation in how the Dog-to-dog seeing and meeting subtests were conducted, because early in the study period, dogs with a history of intolerance of other dogs were sometimes tested with a fake dog rather than a dog from the adoption floor. However, over the nearly five-year study period with results from 975 dogs tested and released for adoption, evaluators used a fake dog with only four dogs during the seeing subtest and two dogs during the meeting subtest, so we expect use of the fake dog had little effect on our findings for the two Dog-to-dog subtests.

## 5. Conclusions

A low prevalence of concerning and especially dangerous behaviors during the multiple tests and subtests of the behavioral evaluation characterized our study population of 975 dogs evaluated and released for adoption. Nevertheless, for some tests and subtests —food guarding and meeting another dog—display of behavior evaluated as dangerous predicted longer lengths of stay at the shelter. Display of concerning behavior on one test that did not create a situation in which dangerous behavior would occur (seeing another dog at a distance) fell just short of being a significant predictor of longer lengths of stay at the shelter. We suspect the longer lengths of stay for dogs that displayed challenging behaviors at the time of evaluation reflect a smaller pool of potential adopters, perhaps resulting from one or more of the following: signage on dog cubicles, direct interactions between potential adopters and dogs, conversation-based discussions between shelter staff and potential adopters when all available information about a dog is disclosed, or dog meets. Understanding the relationships between performance on canine behavioral evaluations and length of stay may aid shelter management of dog populations and help highlight individual dogs requiring special adoption efforts to avoid long shelter stays.

## Figures and Tables

**Table 1:** Demographic data for the 975 dogs included in the study.

Demographic Information	Females	Males
Age class ^1^		
Juvenile	91	92
Adult	333	341
Senior	55	63
Size class (adults and seniors only) ^2^		
Small	122	129
Medium	171	129
Large	95	146
Source		
Surrendered by owner	219	238
Transferred from another shelter	163	159
Picked up as a stray	81	83
Seized by animal control officer	16	16

^1^ Juveniles, from 4 months to <1 year; adults, from 1 year to <8 years; and seniors, ≥8 years. ^2^ Small, <11 kg; medium, 11–24 kg, and large, ≥25 kg; calculated for adults and seniors only because juveniles are still growing.

**Table 2:** Prevalence of concerning or dangerous behavior in dogs during behavioral evaluations at the study shelter (number of dogs showing concerning or dangerous behavior/number of dogs tested and released for adoption).

Test	Concerning Behavior	Dangerous Behavior
Cage presentation: confrontational ^1^		4.1% (40/975)
Cage presentation: friendly ^1^		1.6% (16/975)
Sociability: stand and ignore ^2^	34.7% (338/975)	
Sociability: three strokes ^2^	6.7% (65/975)	
Sociability: sit and ignore ^2^	17.7% (173/975)	
Sociability: pet and talk ^2^	2.8% (27/975)	
Teeth exam ^3^	1.6% (16/975)	2.0% (19/975)
Handling ^3^	11.6% (113/975)	2.7% (26/975)
Arousal	1.6% (16/975)	0.0% (0/975)
Stranger	3.2% (31/975)	2.6% (25/975)
Dog-to-dog: seeing ^2^	3.4% (33/975)	
Dog-to-dog: meeting	13.3% (130/975)	1.7% (16/975)

^1^ Differences between the shelter behavior evaluation form and PetPoint options for data entry led to only dangerous behaviors being included for the two subtests of Cage presentation. ^2^ Dangerous behaviors were not included in the shelter scoring system for the four Sociability subtests and the Dog-to-dog seeing subtest because none of the subtests created situations in which dangerous behaviors were displayed (Table A1). ^3^ Values reflect concerning or dangerous behavior during at least one attempt of the Teeth exam or during at least one subtest of the Handling test because sometimes these tests were cut short for safety reasons.

## Data Availability

The data presented in this study are available in Supplementary Material.

## Supplementary Materials

The following are available online at https://www.mdpi.com/article/10.3390/ani11113272/s1.

## Appendix A

**Table A1:** Behaviors considered concerning or dangerous during canine behavioral evaluations at the study shelter.

Test	Concerning Behaviors	Dangerous Behaviors
1A. Cage presentation: confrontational	Stays in back of kennel or moves there; shaking, low growl, whale eye	Displays defensive or offensive aggression ^1^
1B. Cage presentation: friendly	Stays in back of kennel or moves there; shaking, low growl, whale eye	Displays defensive or offensive aggression
2A. Sociability: stand and ignore ^2^	Makes nonsocial contact for less than 2 s at a time (e.g., sniff); completely ignores evaluator	
2B. Sociability: three strokes ^2^	Exhibits signs of uneasiness (e.g., stiffens); completely ignores evaluator	
2C. Sociability: sit and ignore ^2^	Does not come over; completely ignores evaluator	
2D. Sociability: pet and talk ^2^	Comes over but moves away and never returns; completely ignores evaluator	
3. Teeth exam	Stiffens; exhibits whale eye; whips head; air snaps	Freezes; growls; snarls; snaps; bites
4. Handling	Stiffens; exhibits whale eye; whips head	Freezes; growls; snarls; snaps; bites
5. Arousal	Becomes highly aroused and remains so after play stops	Crosses over to aggression when highly aroused
6. Food bowl and 7. Possession	Stiffens; exhibits whale eye; snarls	Freezes; growls; lunges; snaps; bites Assess-a-hand
8. Stranger	Alarm barks; hackles up; growls; does not calm readily; is eventually friendly upon solicitation but in a cautious way	Alarm barks; hackles up; growls; cannot settle; either will not approach upon solicitation or not safe to allow approach
9A. Dog-to-dog: seeing ^2^	Reacts with threats (barking, growling, snarling, lunging)	
9B. Dog-to-dog: meeting	Displays defensive aggression	Displays offensive aggression

^1^ Defensive aggression: while moving away, barks, growls, bares teeth, shows fearful body postures (e.g., head lowered, tail tucked). Offensive aggression: while moving forward, barks, growls, bares teeth, shows offensive body postures (e.g., direct eye contact, ears erect, tail high). ^2^ Dangerous behaviors were not included in the shelter scoring system for the four subtests of the Sociability test and the Dog-to-dog seeing subtest because these subtests do not create situations in which dangerous behaviors are displayed.
